# Risk Factors for Community-Dwelling Older Adults Dropping Out of Self-Guided, Remote, and Web-Based Longitudinal Research: Predictive Modeling of Data from the Web-LABrainS Platform

**DOI:** 10.2196/68826

**Published:** 2025-11-13

**Authors:** Luke Daniel Braun, H Raymond Allen, Jeffrey N Keller

**Affiliations:** 1 Pennington Biomedical Research Center, Institute for Dementia Research and Prevention, 6400 Perkins Rd, Baton Rouge, LA, 70808, United States, 1 2258920464; 2 Pennington Biomedical Research Center, Computing Services, Baton Rouge, LA, United States

**Keywords:** community-dwelling, longitudinal study, older adult, web-based assessment, study attrition

## Abstract

**Background:**

Little is currently known regarding the feasibility of using a self-guided, remote, web-based platform as the basis for a longitudinal study of aging in community-dwelling older adults (OAs). This study describes the feasibility and risk factors for participant dropout found when using this approach as part of the web-based Louisiana Aging Brain Study (web-LABrainS).

**Objective:**

This study used data from 402 participants in the web-LABrainS effort to determine the feasibility of using a self-guided, remote, and web-based platform as the basis for conducting longitudinal research in community-dwelling older adults. Additionally, we sought to determine the risk factors associated with participant dropout over a 12-month period in web-LABrainS and determine whether the same risk factors associated with dropout from in-clinic longitudinal studies were observed in web-LABrainS dropouts.

**Methods:**

Participants were enrolled in web-LABrainS on a rolling basis using word-of-mouth promotional efforts. Participants used the web-LABrainS platform to provide electronic consent, demographic and health information, answer questionnaires, and complete assessments as part of a self-guided and web-based effort off-site of the clinic (remote). Following completion of the baseline battery, participants were contacted by email every 6 months to complete another round of the web-LABrainS battery. The data in this study were collected from 402 participants, 217 (54.0%) of whom completed baseline, 6-month, and 12-month assessments (adherent participants) and 185 (46%) of whom participated in only the baseline and no subsequent web-LABrainS batteries (dropout participants).

**Results:**

Our study indicates that even with limited participant outreach and retention efforts, it is feasible to conduct longitudinal clinical research studies in community-dwelling OAs using a self-guided, remote, and web-based approach. In contrast to traditional in-clinic longitudinal studies, dropouts from web-LABrainS were not observed to be significantly different with respect to age, education, gender, marital status, or living alone (*P=*.67, .16, .29, .051, .31). Similar to traditional longitudinal studies, dropouts from web-LABrainS had significantly higher use of depression medication, decreased self-reported mobility, and decreased delayed recall performance (*P=*.007, .007, .004). Interestingly, no differences in technology use, comfort with technology, time of assessment, or consent to be contacted about future research were observed between adherents and dropouts (*P=*.17, .36, .47, .40). Predictive binary logistic regression yielded a moderately accurate model and further supported a negative association between cognitive ability and dropout (OR 0.77, 95% CI 0.61-0.96).

**Conclusions:**

Our study provides some of the first clinical evidence for the feasibility of conducting longitudinal human research using a self-guided, remote, and web-based approach. Additionally, these data highlight the similarities and differences in key factors associated with participant dropout using this type of approach compared to traditional longitudinal study formats. The findings from this study may help guide the design and deployment of future longitudinal studies of older adults focused on self-guided, remote, or web-based approaches.

## Introduction

Longitudinal studies of aging have proven to be invaluable in helping to identify the underlying clinical factors involved in the onset and progression of a diverse array of chronic conditions including dementia, frailty, and cardiovascular disease [1-4]. The vast majority of human longitudinal studies conducted to date have focused on the use of in-clinic study measures and the administration of in-person pencil-and-paper assessments and questionnaires [5]. There are multiple advantages to focusing on traditional onsite administration of assessments and measures for longitudinal studies. For example, traditional onsite evaluations significantly increase the control of the study environment, increase the rigor of assessments, and promote the centralization of clinical expertise and resources. There are also multiple disadvantages to focusing on the use of onsite visits for longitudinal human studies. Disadvantages include the high cost of conducting studies in a clinical setting and the limited availability of study visit slots within the typical daily clinic workflow. Additionally, the reliance on in-clinic visits selects for those participants who have the resources and flexibility to travel to the clinic (socioeconomic and geographical burden) and increases the overall entropy for participants to participate in clinical research.

The dropout rate for in-clinic longitudinal studies involving OAs has been reported to range from 15% to 60% [6-23]. Previous studies have identified a diverse array of risk factors associated with participant dropout from in-clinic longitudinal studies [6-23]. Factors reported to drive participant dropout in traditional longitudinal studies include advanced age, lower education, gender, and the presence of chronic health conditions [6-23].

In the last decade, there has been a dramatic increase in the development, validation, and use of self-guided, remote, and web-based assessments as part of clinical research [24-33]. While these efforts preceded COVID-19, the COVID-19 pandemic introduced an urgency and awareness for the need to quickly develop viable alternatives for conducting large-scale clinical research efforts independent of traditional research settings [34-38]. Together, these novel approaches have focused on the development of self-guided or web-based assessments for the collection of demographics, health information, and delivery of questionnaires and assessments outside of the clinic. As a consequence, today there is a growing scientific literature outlining self-guided, remote, or web-based assessments for cognitive, mobility, and mental health studies in older adults (OAs).

Very little is currently known in terms of the feasibility of using a self-guided, remote, and web-based platform for conducting longitudinal research in OAs. Previously, we validated a self-guided, remote, and web-based platform for the collection of demographics, health information, and delivery of questionnaires and clinical assessments [39]. We then initiated efforts to determine the feasibility of using this platform to conduct a self-guided, remote, and web-based longitudinal aging study in community-dwelling OAs. This study was defined as the web-based Louisiana Aging Brain Study (web-LABrainS).

In this research effort, we report on the feasibility of deploying web-LABrainS as a potential format for conducting longitudinal research in community-dwelling OAs. Feasibility was evaluated using extremely limited outreach (word-of-mouth) and limited retention efforts (2 email reminders to encourage participation) in order to define performance using minimal support conditions. We observed that even in this restricted paradigm, the web-LABrainS platform was a feasible option for conducting an initial 12-month period research follow-up. We also observed similarities and differences between the factors associated with study dropout in web-LABrainS, as compared to previous studies using traditional approaches for longitudinal research. Additionally, we describe the correlations between technology use, comfort with technology, time of day for assessments, and other novel findings toward adherence in web-LABrainS. Taken together, these efforts allowed us to achieve our goal of determining the feasibility of using a largely automated, self-guided, and web-based approach for onboarding, assessing, reporting, and conducting follow-up assessments as part of longitudinal studies involving community-dwelling OAs.

## Methods

### Study Participants

All data in the current study were obtained from participants enrolled in web-LABrainS. The web-LABrainS study is a self-guided, web-based battery of questionnaires (demographics, mobility, medication use, and health history) and assessments [39]. Web-LABrainS has been continually enrolling participants since 2020. Participants who heard about web-LABrainS via word-of-mouth (no promotions or outreach efforts for web-LABrainS have been conducted) contacted the Pennington Biomedical Research Center (PBRC) to be enrolled. Participants were then sent an email containing a hyperlink that took them to the web-LABrainS site to consent to and complete the web-LABrainS battery. Individuals were sent an additional email and hyperlink every 6 months to repeat the web-LABrainS battery.

Because subjects enroll in and complete the web-LABrainS battery of assessments on a rolling basis, for the purposes of this analysis, we pulled participant data from our servers on October 17, 2024. Data from participants who enrolled in the study any time after this date, as well as assessment data from participants enrolled in the study, were not included in this analysis.

### Dropout and Adherent Classification

Subjects were classified as either dropouts or adherents of web-LABrainS. A participant was classified as a dropout if they completed their baseline assessment and had no subsequent assessments. A minimum of 9 months had to have passed since their baseline assessment for a participant to be considered a dropout. If a participant completed a second assessment—even if it was more than 9 months after their baseline assessment—they were not classified as a dropout. Participants were classified as adherents if they completed assessments at a minimum of 3 time points (baseline, 6-month, and 12-month assessments). Participants were recruited to the study via minimal outreach efforts that largely relied on word-of-mouth and grassroots promotion by the participants in web-LABrainS. Participants were retained in the study by equally minimal retention efforts that were based on 2 email prompts for those individuals who did not open the email link to complete their web-LABrainS assessment at baseline, 6, or 12 months postbaseline. This approach was undertaken to evaluate the feasibility in a low recruitment and retention environment.

### Components of Web-LABrainS

The components of the web-LABrainS assessment have been described previously in a study of the feasibility of this self-guided, web-based paradigm [39]. We will briefly summarize the components of the web-LABrainS assessments used in this investigation. First, participants were presented with demographic questions about their date of birth, gender identification, racial and ethnic background, marital status, and education. Participants were then asked about their living situation (including whether they live alone and their type of residence), the types of technology they use, their self-rated comfort with technology, as well as their driving status and frequency. Participants are asked about how they rate their own mobility and are given a short questionnaire adapted from the Life-Space Assessment [40]. Participants proceed to provide their level of concern for their memory and whether they rate it worse than others their age. Participants are asked about the number and type of prescription medications they use, and their ability to read a medication label. They are asked to provide their personal medical history including identifying the presence of current chronic health conditions. To conclude the survey portion of the web-LABrainS battery, participants are asked whether they wish to be contacted for research portions in the future.

In the next section of the web-LABrainS battery, participants take multiple validated cognitive assessments, including an 8-item orientation assessment as well as 4-item immediate and delayed recall assessments. Finally, they take an assessment of acute symptoms of depression and anxiety.

### Statistical Analysis

We used 2 types of statistical analysis in this study. We first implemented a between-group comparison of dropouts and adherents. This involved 1-way chi-square tests of independence to assess the significance of the differences in the observed and expected frequencies of all categorical dependent variables that were examined. It also involved Mann-Whitney *U* tests to assess the significance of the differences in the distributions of all numerical-dependent variables that were examined. Exact *P* values were reported; however, a significance threshold (*α*) of .05 was used to report statistically significant results.

Our second form of statistical analysis was a binary logistic regression model, a predictive model trained on our data from which we calculated odds ratios (ORs) and CIs to determine the relative strength of our examined variables as predictors of dropout status. We trained and cross-validated 4 different models. We used different sets of predictors in each of these models and iteratively tuned their hyperparameters for optimal performance. Depending on the model, this involved adjusting the optimization algorithm, regularization method, regularization strength, size trim tolerance for predictor coefficients, convergence tolerance, or number of iterations over multiple runs of the model and selecting that which resulted in the strongest classification performance. We assessed our model’s classification ability using k-fold cross-validation. We first shuffled the dataset and split it into 5 separate folds. Four of the folds were used to train the model, while the last fold was withheld to be used as the test set. This process was repeated with each fold being used as the test set once. The trained model was used to predict the outcomes of the unseen subjects in each set. For each iteration of the model, we generated a classification report that included the model’s precision, recall, and *F*_1_-scores to evaluate model performance for each class (dropout and adherent). The weighted and unweighted averages of these scores across the 2 classes were also provided along with the total model accuracy. The support values for these averages were also included. The 5 classification reports produced for each iteration were then averaged.

We then selected the best-performing model from the cross-validation phase. Of the 4 models tested, our chosen model was tied for the highest overall accuracy but exhibited more consistent performance across validation folds and better classification of the minority class. This model included the variables that significantly differed between groups from our first analysis (self-rated mobility score, delayed recall performance, total number of prescription medications taken, use of depression medication, and quality-of-life self-rating) along with demographic variables (age, race, ethnicity, gender, and years of education). With that subset of data selected, we preprocessed our data in the same way we did during our cross-validation stage. This involved applying 1-hot encoding to all categorical variables, converting them into a binary format that the model was able to use. It also involved converting all continuous variables to a standard scale with a mean of 0 and SD of 1 to ensure equal contribution of these variables to our model. Finally, we added a constant term to the dataset to be used as a reference by the model.

The model itself was based on a logit function. The coefficients of this function were estimated using a convex optimization algorithm with L1 or Lasso (Least Absolute Shrinkage and Selection Operator) regularization. We applied a regularization strength of 0.01 to the coefficient estimation. We also set a trim tolerance of 0.05 for coefficients with a small absolute value. After the model was fit to the full dataset, we retrieved the coefficients of each predictor and calculated the corresponding ORs. The ORs show how the probability of the outcome—in this case, dropping out— changes with an increase of 1 SD to a numerical variable or the presence of a binary variable.

All statistical analysis was performed in Python [41] using functions from the *numpy*, *scipy*, *sklearn*, and *statsmodels* packages [42-45]. Outputs from the Web-LABrainS tool were stored in data structures and manipulated using objects and functions from the *pandas* Python package [46]. Data visualization was accomplished using the *matplotlib* and *seaborn* packages [47,48].

### Ethical Considerations

All study procedures were approved by the PBRC Institutional Review Board (IRB). Informed consent was provided for all participants prior to the initiation of study procedures. Participants were informed that participation was voluntary and that they could opt out at any time. Informed consent included the ability of PBRC researchers to conduct secondary analysis without additional consent. All data were stored in a deidentified manner to maintain participant confidentiality. No compensation was provided for study participation as outlined in the PBRC IRB-approved study protocol. All data used for this study were obtained from web-LABrainS. IRB approval for the web-LABrainS study was obtained from the PBRC IRB (FWA # 00006218) prior to the initiation of web-LABrainS research efforts. The PBRC IRB approval number is 2020-044-PBRC Web-LABrainS. The study complied with ethical standards outlined in the Belmont Report and Declaration of Helsinki.

## Results

In this study, a total of 402 OA participants in web-LABrainS were examined. The demographics of all 402 OA participants at their baseline assessment are provided in Table 1. The baseline age of the entire group was 65.3 years (SD 11.6 years). In terms of representation of different races, genders, and levels of education, our overall sample was overwhelmingly White (355/402, 88.3%), female (298/402, 74.1%), and highly educated (mean 16.8, SD 2.5 years of education). Of the 402 participants in this study, a total of 217 participants (54.0%) completed the web-LABrainS battery at baseline, 6 months, and 12 months. These participants were classified as adherent participants. The remaining 185 (46.0%) completed only the baseline web-LABrainS assessment. These participants were referred to as the dropouts.

**Table 1.** Demographics and between-group comparisons for dropouts and adherents.

Measure	Total, N=402	Dropout, n=185	Adherent, n=217	*P* value
Year of baseline assessment, n (%)				.27
2021	100 (24.9)	44 (23.8)	56 (25.8)	
2022	68 (16.9)	33 (17.8)	35 (16.1)	
2023	231 (57.5)	105 (56.8)	126 (58.1)	
2024	3 (0.7)	3 (1.6)	0 (0.0)	
Time of baseline assessment, mean (SD) (HH:MM)	14:00 (4:00)	14:12 (04:00)	13:48 (04:00)	.47
Age (years), mean (SD)	65.3 (11.6)	65.5 (12.0)	65.1 (11.2)	.67
Age quintiles, n (%)				.52
<50	54 (13.4)	27 (14.6)	27 (12.4)	
50‐60	64 (15.9)	28 (15.1)	36 (16.6)	
60‐70	127 (31.6)	54 (29.2)	73 (33.6)	
70‐80	117 (29.1)	53 (28.6)	64 (29.5)	
>80	40 (10.0)	23 (12.4)	17 (7.8)	
Gender, n (%)				.29
Men	103 (25.6)	42 (22.7)	61 (28.1)	
Women	298 (74.1)	143 (77.3)	155 (71.4)	
Prefer to self-describe	1 (0.2)	0 (0.0)	1 (0.5)	
Race, n (%)				.27
White	355 (88.3)	157 (84.9)	198 (91.2)	
Black or African American	30 (7.5)	19 (10.3)	11 (5.1)	
Asian	6 (1.5)	4 (2.2)	2 (0.9)	
American Indian or Alaska Native	0 (0.0)	0 (0.0)	0 (0.0)	
Other	6 (1.5)	3 (1.6)	3 (1.4)	
Ethnicity, n (%)				.16
Hispanic or Latino	5 (1.2)	2 (1.1)	3 (1.4)	
Non-Hispanic	344 (85.6)	165 (89.2)	179 (82.5)	
Other	53 (13.2)	18 (9.7)	35 (16.1)	
Marital status, n (%)				.05
Married	257 (63.9)	106 (57.3)	151 (69.6)	
Never married	28 (7.0)	11 (5.9)	17 (7.8)	
Common-law partner	5 (1.2)	4 (2.2)	1 (0.5)	
Divorced	70 (17.4)	39 (21.1)	31 (14.3)	
Widowed	39 (9.7)	23 (12.4)	16 (7.4)	
Years of education, mean (SD)	16.8 (2.5)	16.6 (2.6)	16.9 (2.5)	.16
Highest level of education, n (%)				.35
High school or GED	15 (3.7)	10 (5.4)	5 (2.3)	
Some college	68 (16.9)	35 (18.9)	33 (15.2)	
Associate’s degree	20 (5.0)	7 (3.8)	13 (6.0%	
Bachelor’s degree	126 (31.3)	60 (32.4)	66 (30.4)	
Master’s degree	125 (31.1)	52 (28.1)	73 (33.6)	
Doctorate degree	48 (11.9)	21 (11.4)	27 (12.4)	
Living situation, n (%)				.31
Living alone	96 (23.9)	49 (26.5)	47 (21.7)	
Not living alone	306 (76.1)	136 (73.5)	170 (78.3)	
Housing, n (%)				.74
Single residence house	369 (91.8)	168 (90.8)	201 (92.6)	
Assisted living	1 (0.2)	1 (0.5)	0 (0.0)	
Apartment complex	13 (3.2)	7 (3.8)	6 (2.8)	
Stand- alone apartment	5 (1.2)	3 (1.6)	2 (0.9)	
Other	14 (3.5)	6 (3.2)	8 (3.7)	
Average technologies used, mean (SD)	4.0 (1.0)	3.9 (1.1)	4.1 (0.9)	.17
Individual technologies used, n (%)				
Smartphone use	390 (97.0)	179 (96.8)	211 (97.2)	≥.99
Tablet use	307 (76.4)	135 (73.0)	172 (79.3)	.17
Laptop use	342 (85.1)	156 (84.3)	186 (85.7)	.80
Desktop use	330 (82.1)	148 (80.0)	182 (83.9)	.38
Wearable use	252 (62.7)	109 (58.9)	143 (65.9)	.18
Comfort with computers score, mean (SD)				
Comfort with computers score, 1‐5	3.8 (1.5)	3.8 (1.5)	3.9 (1.5)	.36
Comfort with computers, n (%)				.58
Very comfortable	212 (52.7)	92 (49.7)	120 (55.3)	
Slightly comfortable	54 (13.4)	28 (15.1)	26 (12.0)	
I’m okay	63 (15.7)	29 (15.7)	34 (15.7)	
Slightly uncomfortable	9 (2.2)	6 (3.2)	3 (1.4)	
Very uncomfortable	64 (15.9)	30 (16.2)	34 (15.7)	
Mobility, mean (SD)				
Mobility self-score, 1‐5	3.8 (1.0)	3.7 (0.9)	3.9 (0.9)	.007
Life-space mobility score, 0‐6	3.7 (1.2)	3.7 (1.1)	3.7 (1.2)	.96
Falls in last year, n (%)				.86
Fall	80 (19.9)	38 (20.5)	42 (19.4)	
No Fall	322 (80.1)	147 (79.5)	175 (80.6)	
Driving status, n (%)				.12
Regularly drive	383 (95.3)	173 (93.5)	210 (96.8)	
Occasionally drive	16 (4.0)	9 (4.9)	7 (3.2)	
Rarely drive	0 (0.0)	0 (0.0)	0 (0.0)	
Do not drive	3 (0.7)	3 (1.6)	0 (0.0)	
Driving frequency, mean (SD)				
Driving frequency self-score	4.4 (0.7)	4.4 (0.8)	4.5 (0.6)	.28
Cognition, mean (SD)				
Orientation score, 0‐4	7.8 (0.4)	7.8 (0.4)	7.8 (0.4)	.42
Immediate recall score, 0‐4	4.0 (0.1)	4.0 (0.1)	4.0 (0.1)	.53
Delayed recall score, 0‐4	3.8 (0.6)	3.7 (0.7)	3.9 (0.5)	.004
Memory compared to others their age, n (%)				.30
Worse	77 (19.2)	40 (21.6)	37 (17.1)	
Not worse	325 (80.8)	145 (78.4)	180 (82.9)	
Memory concern score, mean (SD)				
Memory concern self-score	1.8 (0.6)	1.8 (0.6)	1.8 (0.6)	.42
Memory concern, n (%)				.22
Extremely concerned	4 (1.0)	1 (0.5)	3 (1.4)	
Very concerned	32 (8.0)	20 (10.8)	12 (5.5)	
Some concern	253 (62.9)	113 (61.1)	140 (64.5)	
No concern	113 (28.1)	51 (27.6)	62 (28.6)	
Depression, mean (SD)				
Depression score, 0‐64	12.7 (9.8)	13.0 (9.6)	12.5 (10.0)	.39
Quality of life, mean (SD)				
Quality of life self-score	79.0 (16.5)	76.5 (17.2)	81.1 (15.5)	.003
Future contact for research, n (%)				.40
Consented	387 (96.3)	176 (95.1)	211 (97.2)	.
Did not consent	15 (3.7)	9 (4.9)	6 (2.8)	
Total prescription medications, mean (SD)	3.4 (2.7)	3.9 (2.9)	2.9 (2.4)	.001
Prescription medication types, n (%)				
Acid suppression	92 (22.9)	49 (26.5)	43 (19.8)	.14
Cholesterol	181 (45.0)	86 (46.5)	95 (43.8)	.66
Diabetes	39 (9.7)	22 (11.9)	17 (7.8)	.23
Sleep aids	83 (20.6)	46 (24.9)	37 (17.1)	.07
Depression	78 (19.4)	47 (25.4)	31 (14.3)	.007
Anxiety	90 (22.4)	47 (25.4)	43 (19.8)	.22
Medical conditions, n (%)				
Diabetes	31 (7.7)	16 (8.6)	15 (6.9)	.64
High blood pressure	185 (46.0)	93 (50.3)	92 (42.4)	.14
High cholesterol	197 (49.0)	92 (49.7)	105 (48.4)	.87
Thyroid deficiency	80 (19.9)	42 (22.7)	38 (17.5)	.24
Cancer	65 (16.2)	28 (15.1)	37 (17.1)	.70
Alcohol abuse	19 (4.7)	11 (5.9)	8 (3.7)	.41
Anxiety	102 (25.4)	49 (26.5)	53 (24.4)	.72
Stroke	8 (2.0)	2 (1.1)	6 (2.8)	.40
B12 deficiency	27 (6.7)	13 (7.0)	14 (6.5)	.98
Sleep apnea	66 (16.4)	28 (15.1)	38 (17.5)	.61
Depression	121 (30.1)	63 (34.1)	58 (26.7)	.14
Concussion or TBI	10 (2.5)	6 (3.2)	4 (1.8)	.56
TIA	7 (1.7)	3 (1.6)	4 (1.8)	≥.99
Atrial fibrillation	21 (5.2)	10 (5.4)	11 (5.1)	≥.99
Neurological disease	13 (3.2)	8 (4.3)	5 (2.3)	.39
Heart attack	9 (2.2)	6 (3.2)	3 (1.4)	.36
Drug abuse	4 (1.0)	1 (0.5)	3 (1.4)	.73
Parkinson disease	0 (0.0)	0 (0.0)	0 (0.0%	≥.99

Our between-group comparison identified that there were many metrics in which there were no significant differences between dropout and adherent participants. These included basic demographic variables such as average age, distribution of participants within age quintiles, gender, race, ethnicity, average years of education, and highest level of education (*P*=.67, .52, .29, .27, .16, .16, and .35). Differences in marital statuses neared significance (*P*=.051) but were not significant. Dropouts and adherents had statistically similar frequency of living alone, housing situations, driving statuses, and self-rated driving frequency (*P*=.31, .74, .12, .28). Results from the Life Space mobility scale did not significantly differ between groups (*P*=.96). Next, we sought to compare the 2 groups in terms of technology use and comfort with technology. Self-rated comfort with computers and average comfort with computers score (*P*=.58 and *P*=.36), average number of technologies used (*P*=.17) as well as the frequency of smartphone, tablet, laptop, desktop, and wearable technology use (*P*=*P*≥.99, *P*=.17, *P=*.80, *P*=.38, *P*=.18) were not significantly different between the 2 groups. Cognitively, dropouts and adherents did not display significantly different performance on our orientation or immediate recall assessments (*P*=.42, .53). There was also not a significant difference in depressive symptoms between groups (*P*=.39). There were no significant between-group differences in whether participants rated their memory as worse than others their age and in their self-rated memory concern and average memory concern score (*P*=.30, .22, .42) Use of medications for acid suppression, cholesterol, diabetes, sleep aid, and anxiety did not differ significantly between groups (*P*=.14, .66, .23, .07, .22). Medical history of diabetes, high blood pressure, high cholesterol, thyroid deficiency, cancer, alcohol abuse, anxiety, stroke, B12 deficiency, sleep apnea, depression, concussion or traumatic brain injury, transient ischemic attack, atrial fibrillation, neurological disease, heart attack, drug abuse, and Parkinson disease did not differ significantly between groups (*P*=.64, .14, .87, .24, .70, .41, .72, .40, .98, .61, .14, .56, ≥.99, .≥99, .39, .36, .73, ≥.99). Interestingly, consent to be contacted for future research did not differ significantly between groups (*P*=.40).

The metrics in which we observed differences between dropout and adherent groups ranged across multiple domains of health. The dropout group had significantly lower self-rated mobility scores (*P*=.007), significantly lower delayed recall scores (*P*=.004), higher total prescription medications taken (*P*=.001), higher self-reported use of depression medication (*P*=.007), and lower self-rated quality of life (*P*=.003) as compared to the adherent group.

With initial findings from our between-group comparisons, we proceeded to the modeling stage of our analysis. We constructed 4 binary logistic regression models consisting of different sets of predictors chosen based on the results of the between-group tests. The footnote of Table 2 defines the predictor set of each model. Hyperparameters were tuned for maximum classification strength according to the protocols described in the Methods section above. Ultimately, Model 3, a model with a predictor set consisting of all significant between-group variables and basic demographic variables, was tied for the highest overall accuracy but exhibited more consistent performance across validation folds and better classification of the minority class. The classification metrics from the full model comparison are shown in Table 2.

**Table 2.** Binary logistic regression model performance in the cross-validation stage.

	Model 1^a^	Model 2^b^	Model 3^c^	Model 4^d^
Adherent classification metrics, mean (SD)				
Precision	0.59 (0.06)	0.61 (0.05)	0.61 (0.04)	0.57 (0.03)
Recall	0.64 (0.05)	0.76 (0.09)	0.73 (0.07)	0.7 (0.08)
*F*_1_-score	0.61 (0.05)	0.68 (0.06)	0.67 (0.05)	0.63 (0.04)
Support	54.25 (0.5)	54.25 (0.5)	54.25 (0.5)	54.25 (0.5)
Dropout classification metrics, mean (SD)				
Precision	0.53 (0.09)	0.61 (0.1)	0.6 (0.07)	0.53 (0.06)
Recall	0.48 (0.11)	0.44 (0.07)	0.46 (0.05)	0.39 (0.06)
*F*_1_-score	0.5 (0.1)	0.51 (0.07)	0.52 (0.06)	0.45 (0.04)
Support	46.25 (0.5)	46.25 (0.5)	46.25 (0.5)	46.25 (0.5)
Classification metrics across classes, mean (SD)				
Unweighted average precision	0.56 (0.07)	0.61 (0.07)	0.6 (0.06)	0.55 (0.04)
Unweighted average recall	0.56 (0.07)	0.6 (0.06)	0.6 (0.05)	0.55 (0.03)
Unweighted average *F*_1_-score	0.56 (0.08)	0.59 (0.07)	0.59 (0.05)	0.54 (0.03)
Unweighted average support	100.5 (0.58)	100.5 (0.58)	100.5 (0.58)	100.5 (0.58)
Weighted average precision	0.56 (0.07)	0.61 (0.07)	0.61 (0.05)	0.55 (0.04)
Weighted average recall	0.56 (0.07)	0.61 (0.07)	0.61 (0.05)	0.56 (0.04)
Weighted average *F*_1_-score	0.56 (0.07)	0.6 (0.06)	0.6 (0.05)	0.55 (0.03)
Weighted average support	100.5 (0.58)	100.5 (0.58)	100.5 (0.58)	100.5 (0.58)
Overall model accuracy, mean (SD)				
Accuracy	0.56 (0.07)	0.61 (0.07)	0.61 (0.05)	0.56 (0.04)

a Model with predictor set consisting of all nonredundant metrics collected, L1 regularization with convex optimization algorithm, regularization strength of 0.01, size trim tolerance of 0.1 for coefficients, convergence tolerance of 1×10−10, 100 iterations.

b Model with predictor set consisting of only variables significant in between-group comparison, Newton-Raphson root-finding algorithm for optimization, convergence tolerance of 1×10−10, 100 iterations.

c Model with predictor set consisting of variables significant in between-group comparison and basic demographic variables, L1 regularization with convex optimization algorithm, regularization strength of 0.01, size trim tolerance of 0.05 for coefficients, convergence tolerance of 1×10−10, 100 iterations.

d Model with predictor set consisting of variables significant in between-group comparison and basic demographic variables as well as all second-degree interactions, L1 regularization with convex optimization algorithm, regularization strength of 0.01, size trim tolerance of 0.1 for coefficients, convergence tolerance of 1×10−10, 100 iterations.

Our logistic regression model, based primarily upon these significantly different between-group findings, showed several predictors with varying influence on the odds of the dropout (Table 3). Older age, identifying as female, having a Black or African American or Asian racial background, and the use of depression medication were all found to be associated with slightly increased odds, but the CIs for each encompass 1, indicating limited significance. Having a higher delayed recall was the most notable of the significant associations we found (OR 0.77, 95% CI 0.61-0.96), suggesting that higher delayed recall ability might reduce the odds of dropping out. Additionally, taking more prescription medications shows a significant association with an increase in the odds of dropping out (OR 1.3, 95% CI 1.01-1.68). Identifying as an ethnicity other than Hispanic or Latino or non-Hispanic was also shown to reduce dropout odds (OR 0.78, 95% CI 0.62-0.97). Overall, while some predictors hint at associations, few reach strong statistical significance.

**Table 3.** Binary logistic regression model results.

Predictor	Coefficient	Odds ratio (95% CI)
Constant term	−0.18	0.83 (0.62-1.12)
Age	0.13	1.14 (0.9-1.44)
Gender		
Women	0.13	1.14 (0.91-1.43)
Prefer to self-describe	−0.34	0.71 (0.01-51.46)
Race		
Black	0.17	1.18 (0.95-1.47)
Asian	0.2	1.22 (0.98-1.52)
Other	0.06	1.06 (0.84-1.34)
Two or more races	Trimmed	N/A^a^
Ethnicity		
Hispanic or Latino	−0.05	0.95 (0.77-1.17)
Other	−0.25	0.78 (0.62-0.97)
Years of education	−0.07	0.93 (0.75-1.15)
Self-rated mobility score	−0.14	0.87 (0.69-1.11)
Delayed recall score	−0.27	0.77 (0.61-0.96)
Total prescription medications	0.26	1.3 (1.01-1.68)
Use of depression medication	0.17	1.19 (0.95-1.49)
Quality of life	−0.2	0.82 (0.65-1.03)

a Not available.

**Figure 1.**
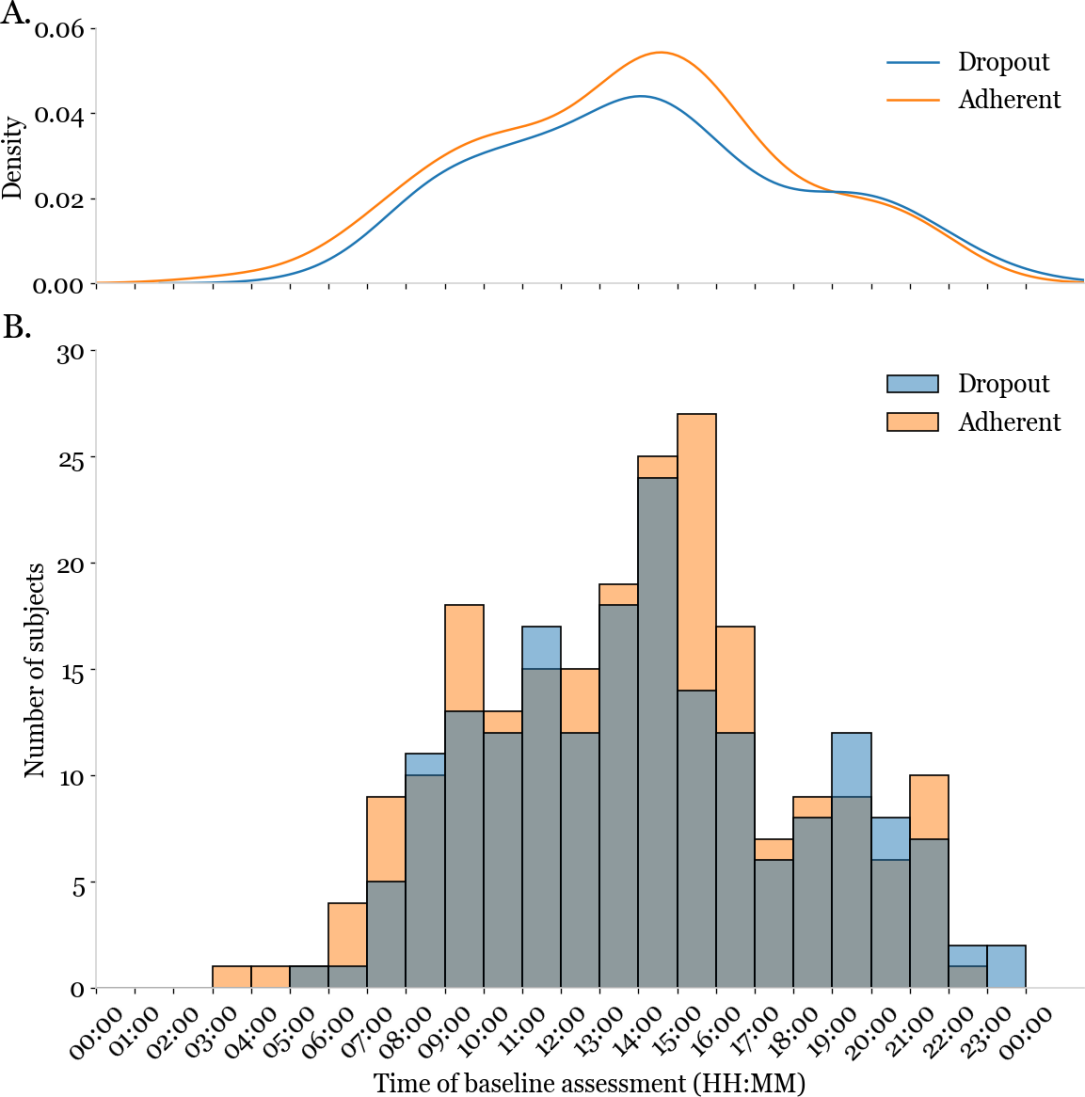
Kernel density estimation function and histogram for web-based Louisiana Aging Brain Study assessment start times. (A) Density plots showing the temporal distribution of baseline assessments for dropout (blue) and adherent (orange) participants. (B) The number of participants who completed their baseline assessment within each hour-long bin in a 24-hour day is depicted in histogram form. Distributions for both dropouts (blue) and adherents (orange) were displayed together at partial opacity, with overlapping parts of the distributions shown in gray. All times were reported relative to each participant’s time zone.

As part of our feasibility efforts, we next sought to determine whether there were significant differences between the time of the day that adherents and dropouts completed their baseline web-LABrainS battery. We observed that dropouts and adherents largely completed their respective assessment batteries at the same times of day (Figure 1). The mean time of day at which all participants completed their assessments was 2:00 PM (SD 4 hours) and the distributions of the times of day at which dropouts and adherents completed their assessments did not differ significantly between groups (Table 1), an indication of the convenience of an online assessment tool that is always available to the user. Interestingly, none of the 402 participants took their assessment battery between midnight and 3:00 AM of the participant’s local time, while all other times of day had some amount of representation.

## Discussion

### Principal Findings

Our results in this study support our initial hypothesis that the use of a self-guided, remote, and web-based battery can be used to conduct longitudinal research in community-dwelling OAs. We identified that demographic characteristics such as age, education, gender, marital status, and living alone were not significantly correlated with participant dropout. Similarly, we observed that the reported comfort level with using technology, the amount of technology use, interest in research, and the time of assessment were also not correlated with participant dropout. Conversely, we identified a diverse array of medical-related issues that were significantly correlated with study dropout, including use of depression medication, decreased mobility, and impairments in delayed recall performance, all of which were significantly correlated with study dropout. The importance of each of these findings is discussed below.

While the level of dropouts in this study was high, it is in line with what has been reported by in-clinic longitudinal studies. It is important to point out that recruitment of participants to web-LABrainS in this feasibility study did not involve efforts to target specific populations or efforts to do large-scale recruitment, being that recruitment was largely restricted to word-of-mouth among participants and a byproduct of correspondence with our institute. Additionally, it should be pointed out that participants received 2 reminder emails to conduct their 6- or 12-month assessment batteries and received limited or no additional follow-up (or forms of follow-up) to maintain participant retention in this feasibility study. Given the extremely restricted outreach and retention efforts in this study, we believe that even though our feasibility effort went out to only 12 months, it shows that this approach is a feasible effort to have community-dwelling OAs maintain participation in a longitudinal research effort that involves repeated assessments over time.

Many in-clinic longitudinal studies have investigated which of the variables collected in their demographic surveys, health questionnaires, and physical or cognitive assessments are significant predictors of participant attrition. One of the most commonly identified variables reported in the literature is age, although some studies claim older age makes a subject more prone to attrition [12,14,18,19,21] while others suggest younger subjects have a greater risk of study dropout [7-9,23]. Past research also shows variability in other predictors of study dropout including gender and education. Several studies identify being male as a significant predictor of study dropout [8,12,21], while others have shown that being female increases the risk for study dropout [16,19]. Likewise, past research has found both low levels of education [7,8,10,15,16,20,22,23] and high levels of education [21] to be significant predictors of study dropout. Taken together, data from this study and existing literature suggest that the retention of participants in longitudinal studies may be impacted by some factors (age, gender, and education) that are study-specific and therefore challenging to control for or successfully address.

Other predictors of participant attrition did not vary in directionality. These variables associated with participant dropout included having lower socioeconomic or job status, lower cognitive function, and lower self-reported or general health [7,10,12,15,19,21,22]. Medical conditions such as chronic stress, cardiovascular disease, coronary artery disease, heart attack, respiratory dysfunction, diabetes, depression, and anxiety were commonly identified predictors of study dropout when surveyed for or directly assessed [9-11,19,22]. Finally, lifestyle factors such as smoking, low physical activity, and low social and community involvement were also commonly associated with study dropout [7,10,12,21]. Living situations played an interesting role in the risk of attrition in previous studies. The presence of familial conflict or young children was identified as a significant predictor of study dropout [9]. Living alone or having fewer potential caretakers also significantly predicted attrition [12,18,19]. Based on the consistency of these findings, future studies should set out to fully address the potential impact of each of these aspects on participant retention. For example, future studies (including web-LABrainS 2.0) should consider incorporating strategies that minimize the negative impacts of lower socioeconomic status (providing participant stipends and maximizing flexibility for when study visits occur), impaired cognition (consider incorporating study partner and simplifying assessment screens), and poor health (allow study staff to assist with evaluation if requested, provide alternative types of assessments for individuals with physical limitations).

Our studies identified several factors associated with dropout in this web-LABrainS feasibility effort and the findings from studies using traditional longitudinal study approaches. For example, we observed that dropout in the web-LABrainS effort was not correlated with age, education, gender, marital status, or living alone. In contrast, dropouts in web-LABrainS had significantly higher levels of depression, decreased self-reported mobility, and decreased memory performance (delayed recall). In addition, we identified 2 novel factors—decreased quality of life and increased number of prescribed medications—that were associated with study dropout. Interestingly, in this web-LABrainS feasibility research effort, no differences in technology use, comfort with technology, or time of assessment were observed between adherent participants and dropouts. Another surprising finding in this study was the finding that there was an extremely high percentage (greater than 95%) of both adherent participants and dropout participants in web-LABrainS who confirmed a willingness to be contacted about future research studies. These data suggest that alternative factors, including the perceived value of participation in the specific study, play a role in participant retention. For example, it is likely that some participants continue in a longitudinal study solely based on personal reasons that have not been discussed above (family member impacted by the disease or dedication to a university, clinician, or researcher). Alternatively, participants may differentially stay in a longitudinal study based on their perceived value of the study results they receive. It should be noted that for web-LABrainS, we routinely receive participant correspondence saying they value the opportunity to participate because of a family member who had dementia or because they highly value the study summaries they receive at the conclusion of each assessment.

### Limitations

There are multiple limitations in this feasibility study. For example, our web-LABrainS participants are not a representative sample of the community, and in future research efforts, it will be critical to optimize outreach such that the recruitment of a representative community sample is reliably obtained. With a more representative sample and larger sample size, we could also expect predictive modeling for risk of dropout to improve in accuracy from the binary logistic regression model shared in this study. Additionally, there was an expected elevated dropout in this web-LABrainS feasibility study due to the extremely limited nature of participant retention efforts (2 email reminders). In future studies, it will be key to identify and implement features of our platform to maximize the retention of study participants. We believe, based on the incidental feedback from web-LABrainS participants over the course of this feasibility study, that the major reason for study participation is the fact that each participant gets a detailed and automated report on their cognitive function at the conclusion of the web-LABrainS assessment. Continuing to optimize feedback and related collaterals to study participants will likely be critical to ensuring the long-term retention of study participants for years or even decades in longitudinal research efforts.

### Conclusions

Taken together, the findings in this study provide a framework for the design and implementation of longitudinal research studies that focus on the use of a self-guided, remote, and web-based approach. Additionally, the data in this study provide important findings on how different demographic, health, and technology use aspects correlate with adherence or dropout in the web-LABrainS approach. In addition to being feasible, we believe using this new paradigm for longitudinal research will significantly decrease participant burden, dramatically lower research costs, and allow for significantly greater recruitment of participants across a wide geographic area. In future studies, it will be critical to develop methodologies and approaches that facilitate participation by a more racially and socioeconomically diverse array of study participants. These efforts will likely include outreach on social media platforms and collaborations with stakeholders in the community that align with racially and socioeconomically diverse populations. It will likely be important in these future efforts to make the outreach and onboarding of participants as frictionless as possible for participants by optimizing the user interface and user experience. In the interest of improving the experience of the community stakeholders involved in the future research effort, optimizing the application programming interface of the research platform will be a high priority. The impact of these efforts will have a significantly greater clinical impact if they can begin to be incorporated into clinical care as screening, patient engagement, and caregiver support tools. Our future efforts will attempt to incorporate each of these aspects as well as develop pathways for web-LABrainS participants to be connected quickly with study staff and health care professionals when mild cognitive impairment or dementia is identified as part of their longitudinal assessment.

## Abbreviations

IRB: institutional review board
Lasso: Least Absolute Shrinkage and Selection Operator
OR: odds ratio
PBRC: Pennington Biomedical Research Center
web-LABrainS: web-based Louisiana Aging Brain Study
